# Language Comprehension in the Balance: The Robustness of the Action-Compatibility Effect (ACE)

**DOI:** 10.1371/journal.pone.0031204

**Published:** 2012-02-21

**Authors:** Rolf A. Zwaan, Nathan van der Stoep, Tulio Guadalupe, Samantha Bouwmeester

**Affiliations:** 1 Department of Psychology, Erasmus University Rotterdam, Rotterdam, The Netherlands; 2 Department of Experimental Psychology, Hemholtz Institute, Utrecht University, The Netherlands; 3 Max Planck Institute for Psycholinguistics, Nijmegen, The Netherlands; Centre de Neuroscience Cognitive, France

## Abstract

How does language comprehension interact with motor activity? We investigated the conditions under which comprehending an action sentence affects people's balance. We performed two experiments to assess whether sentences describing forward or backward movement modulate the lateral movements made by subjects who made sensibility judgments about the sentences. In one experiment subjects were standing on a balance board and in the other they were seated on a balance board that was mounted on a chair. This allowed us to investigate whether the action compatibility effect (ACE) is robust and persists in the face of salient incompatibilities between sentence content and subject movement. Growth-curve analysis of the movement trajectories produced by the subjects in response to the sentences suggests that the ACE is indeed robust. Sentence content influenced movement trajectory despite salient inconsistencies between implied and actual movement. These results are interpreted in the context of the current discussion of embodied, or grounded, language comprehension and meaning representation.

## Introduction

What happens when we understand a sentence like *The boy dove into the pool*? The traditional assumption is that we construct a language-like mental representation, such as [[P1,DOVE, BOY][P2, INTO, POOL, P1]], whereby the first proposition, P1, represents the fact that the boy dove and the second proposition, P2, that the target location of this action was the pool. This representation captures the intuition that the sentence conveys two things about the described situation. However, such representations may be viewed as “a convenient shorthand” of the mental representations actually formed by comprehenders [1]. According to more recent theories of cognition [2]–[4], mental representations can be summarized in language-like propositions for certain research purposes but they should be thought of as mental representations that are grounded in the brain's systems for perception and action. For example, in the case of the example sentence the comprehender might form a visual representation of the pool, a somatosensory representation of cool water suddenly enveloping the body, and a motor representation of the act of diving. In this article, we are concerned with this latter component of a purported sensorimotor representation: the motor component.

Language comprehension and the brain's motor system interact. This has been demonstrated by numerous behavioral and neuroimaging studies, but the exact interpretation of these findings remains unclear. There is, for example discussion as to whether motor activation is necessary for comprehension or whether it is the result of other levels of processing [5]–[7]. To begin tackling this issue, it is useful to revisit what has proved the most influential behavioral evidence regarding the role of motor activation in language comprehension: the action-compatibility effect [8]. How robust is this effect? Does it occur even when the action to be performed by the subject is incompatible on a number of important dimensions with the action described in the text?

In a seminal study [8], subjects made sensibility judgments to sentences (does the sentence make sense yes or no?) by releasing a button and pressing one of two buttons located either closer to the body or farther away from the body. On some critical trials, the sentence described an action that involved moving the hand toward or away from the body. For example, a sentence like *He closed the drawer* describes an action that involves moving the hand away from the body, whereas *He opened the drawer* describes an action that involves moving the hand toward the body. The key finding, which was dubbed the action-compatibility effect (ACE) [8], was that responses were faster when the action described in the sentence was congruent with the action the subject had to perform to respond to the sentence than when the action was incongruent. In further pursuit of this line of thinking, a later study introduced a related paradigm that allowed the researchers to examine the waxing and waning of motor resonance during sentence comprehension (rather than at the end of the sentence) [9]. In this paradigm, subjects read sentences incrementally a few words at a time by rotating a knob, with five degrees of rotation corresponding to the presentation of a new sentence segment. The critical sentences in their experiments all involved manual rotation (e.g., opening a bottle, screwing in a light bulb). The main finding was that ACE occurred during sentence processing and more specifically that its occurrence coincided with linguistic focus on the action [10].

What these ACE studies have in common is that the action performed by the subject is very similar to the actions described in the sentences. For example, turning a knob is similar to opening a bottle. From the standpoint of trying to find constraints on motor activation in language processing, however, it is important to know whether ACE effects are eliminated when the action performed by the subjects is incongruent or even inconsistent with the described action. But how can an ACE be examined if the described action and the performed action are incompatible? The answer resides in the fact that compatibility is not an all-or-none phenomenon. Actions can be decomposed into different components. Take the action described by *He dove into the pool*. This action might have components such as bending the knees, pushing off, putting the arms forward, and putting the head between the arms. This action is clearly incompatible with standing straight up and moving the body slightly to the right, as this is orthogonal to the described direction. However, if the ACE is robust and not task specific, one might still expect a small forward component in this transversal movement. Conversely, one might expect a small backward component if the sentence describes a backward movement, as in *The teenager plopped down on the couch*.

An even stronger incompatibility between described and performed action arises when the starting postures are different. For example, diving normally presupposes that one is standing. So if subjects are seated and moving their body sideways to respond to the sentences, there is a postural and a directional incompatibility between the described and the performed action. Can the ACE survive such a dual incompatibility?

To answer such questions we made use of a novel way to test the ACE. Subjects were standing or seated on the Wii™ balance board and moved to the right or left to indicate if a sentence, presented on a computer monitor, was sensible or not. Because the balance board provides temporally and spatially sensitive information about the body's center of pressure (COP) [11], we were able to measure balance shifts from left to right as well as forward and backward, with the latter shifts being of theoretical importance.

Because one can only lean forward a certain amount before needing to take corrective action, we expected that if the ACE occurs despite the incompatibilities between described and performed actions, sentences describing forward balance shifts would probably not evoke further forward movement, but rather modulate the trajectory of forward movement over time. That is, we expected different anterior-posterior (AP) balance curves over time for the forward and backward conditions.

**Figure 1:**
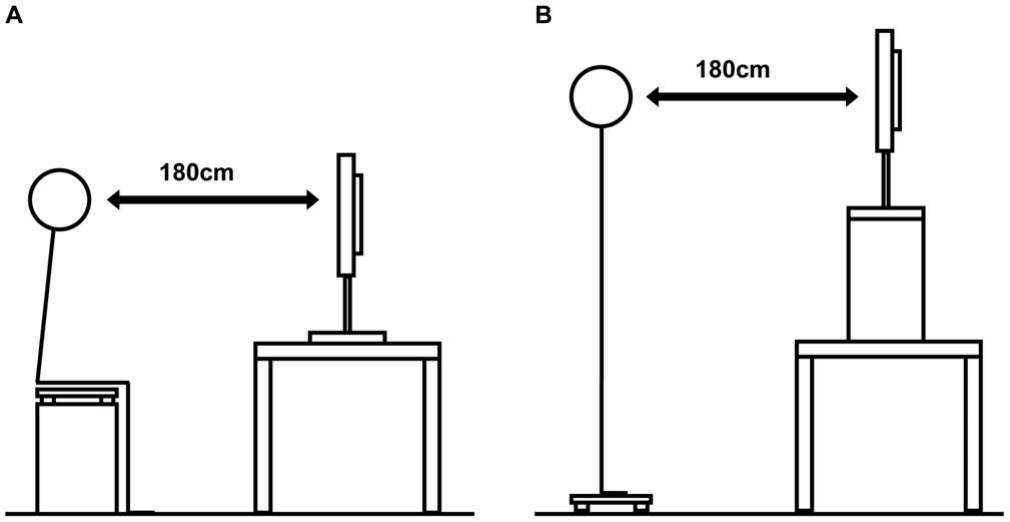
Experimental setup for the experiments. The sitting version is depicted in (A) and the standing version in (B).

**Figure 2:**
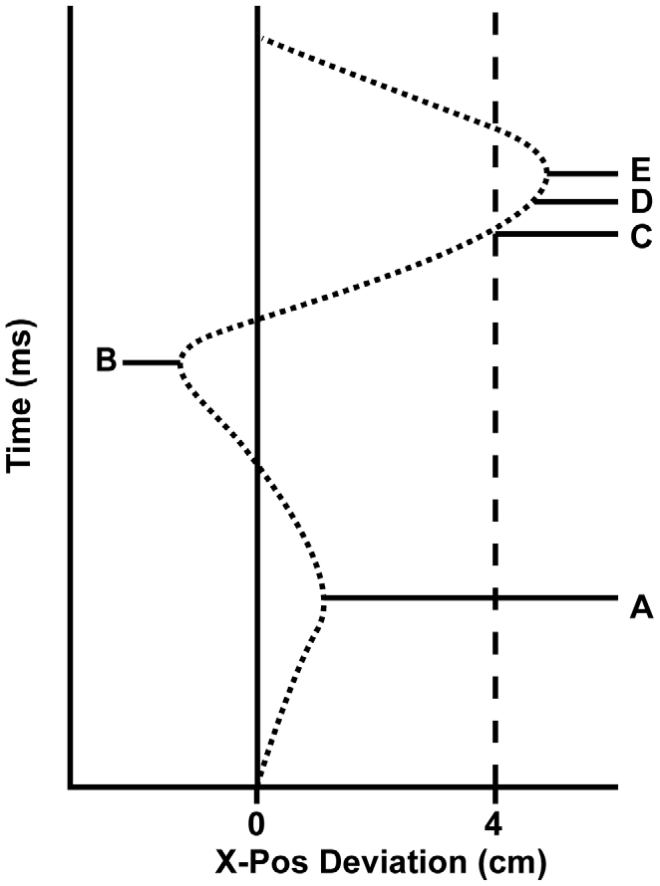
Typical x-axis response curve for leaning to the right (schematic) showing the response onset (time A), the response push-off (time B), the response threshold (time C), the response cut-off 500 ms after the threshold has been exceeded (time D), and the maximum response amplitude (time E).

We conducted two experiments to test this prediction. In one experiment, the subjects were standing on the balance board while moving sideways to judge the sensibility of sentences and in the other experiment the subjects were sitting on the balance board, which had been mounted on a customized piano bench (see Figure 1). We measured the entire trajectory of movement along the forward-backward axis for each trial. We expected to find differences between conditions (forward vs. backward) in the y-axis curves from response onset (time A in Figure 2) to response cut-off (time D in Figure 2) on the x-axis, because we assumed that at this moment the sentence was processed completely. We chose the response cut-off as the endpoint for each curve, because after the response cut-off the sentence disappeared, the fixation cross was shown, and participants moved their COP to the center of the fixation cross.

## Materials and Methods

### Participants

Forty subjects participated for course credit in this study (10 men/30 women). All participants were students at the Erasmus University Rotterdam. They provided oral consent to participating in the experiment. Written consent was not obtained because the experiment was noninvasive. This is in accordance with departmental practice approved by the ethics committee.

### Apparatus and Stimuli

The experiment was presented on a 22-inch monitor (20 inch visible, 100 Hz, Resolution: 1280×1024). In the standing version, a monitor was mounted on a stand on a desk. No stand was used in the sitting version. In both versions, the height of the monitor was adjusted for each participant so that subjects' eye-height was at the same height as the center of the screen. The distance between subjects and the screen was approximately 180 cm (70.9 inch). Thirty-six sentences were presented to each participant in the middle of the screen (letter type: Arial, size: 28). These included 8 sensible sentences in which the action of the agent implied a forward leaning body posture (e.g. *The man petted the little dog*), 8 sensible sentences in which the action of the agent implied a backward leaning body posture (e.g. *The boy looked up at the clock tower*), and 20 filler sentences (sensible and non-sensible). The stimulus sentences are shown in Table 1. To measure body posture, we used a WBB connected to a PC via a Bluetooth connection. Custom-made software was used to present stimuli and record balance data. A custom made bench with a stable flat surface was used to place the WBB on in the sitting version. This bench was adjustable in height.

**Table 1:** Stimulus Sentences: Dutch original (English translation).

**Backward**
De houthakker hief de bijl op. (The lumberjack raised his axe)
De jongen zette zich schrap bij het touwtrekken. (The boy braced himself in the tug of war)
De man deinsde terug voor de stier. (The man recoiled from the bull)
De matroos hees het zeil. (The sailor hoisted the sail)
De tiener plofte neer op de bank. (The teenager plopped down on the couch)
De vrouw gleed uit op het ijs. (The woman slipped on the ice)
Het meisje hield de grote hond in bedwang. (The girl restrained the big dog)
Hij keek omhoog naar de kerkklok. (He looked up to the church bell)
**Forward**
De jongen strikte zijn veter. (The boy tied his shoe lace)
De man aaide de kleine hond. (The man petted the small dog)
De man pakte zijn koffertje op. (The man picked up his briefcase)
De ridder boog voor de koning. (The knight bowed to the king)
De tuinman duwde de kruiwagen. (The gardener pushed the wheelbarrow)
De vrouw plukte de bloem. (The woman picked the flower)
De zwemmer dook in het zwembad. (The swimmer dove into the pool)
Het meisje maakte een koprol voorover. (The girl summersaulted)
**Filler - Nonsensible**
De agent bekeurde de vogel. (The officer gave a ticket to the bird)
De bejaarde bakte de ontkenning. (The elderly person baked the negation)
De jongen verfde de vergissing. (The boy painted the mistake)
De man at de wolk. (The man ate the cloud)
De man besmeerde het geluid. (The man smeared the sound)
De muzikant bespeelde de speld. (The musician played the pin)
De puber schopte de tijd. (The teenager kicked the time)
De slager sneed de gedachte. (The butcher cut the thought)
De vrouw bestrafte de pinda. (The woman punished the peanut)
De vrouw dronk de vrede. (The woman drank the peace)
Het kind sloeg de metafoor. (The child hit the metaphor)
Het meisje droeg de maan. (The girl carried the moon)
**Filler - Sensible**
De atleet deed zijn schoenen uit. (The athlete took off his shoes)
De bakker sloot zijn winkel. (The baker closed his shop)
De jongen hield van films. (The boy liked movies)
De man had een idee voor een boek. (The man had an idea for a book)
De visser had geen aas meer. (The fisherman was out of bait)
De vrouw kocht een auto. (The woman bought a car)
Het kind geloofde in Sinterklaas. (The child believed in Santa)
Het meisje had trek in een ijsje. (The girl wanted an ice-cream)

### Procedure

In the standing version subjects stood on the balance board during the whole experiment and were instructed to hold their arms alongside their body. In the sitting version, before starting the experiment, subjects had to sit straight up in the middle of the WBB that was mounted to the bench. When necessary, the experimenter adjusted the height of the bench to ensure that the knees of a participant were in a 90° angle perpendicular to the floor. Subjects were instructed to place their hands in a comfortable position on their knees. The rest of experimental procedure in the sitting version was equal to standing version.

Before starting the experiment, we calibrated the WBB for each participant so that the center of a fixation cross corresponded with their neutral body posture (sitting or standing straight). Next, participants were instructed to decide whether a sentence was sensible or not by leaning left for non-sensible sentences or right for sensible sentences. The x-axis values of COP of the WBB represent the weight proportion on the left and right sensors, showing subjects' medio-lateral (ML) balance; whereas the y-axis values of the COP represent the weight proportion on the front and back sensors of the WBB showing subjects' anterior-posterior (AP) balance. An x-value of 0 represents a weight distribution of 50% on the left and 50% on the right sensors. Prior to each sentence, a fixation cross was shown. Subjects were instructed to keep their COP within 4 cm^2^ centered on their neutral body posture for 500 ms. During this fixation period, visual feedback was provided on the screen. We defined the COP as the orthogonal projection of the center of gravity on the horizontal plane (the balance board). Positions on the x- and y-axis are expressed in cm distance to the point of reference, which is standing or sitting straight up.

A pilot study revealed a typical x-axis response curve when subjects leaned left or right. Figure 2 shows this curve for leaning to the right. The curve is the same for leaning to the left but mirrored and shows that subjects push themselves off in the opposite direction (at time B) before moving into the intended direction (at time E). A small push-off for the sway at time B is also visible (time A). Responses were defined as exceeding a threshold value of −4 (left) or 4 (right) on the x-axis (time C in Figure 2) for 500 ms (time D in Figure 2). A cut-off of −4 or 4 was chosen, because this way a clear ML balance shift was needed to respond, without maintaining an extreme body posture. This ensured that subjects did not get tired during the course of the experiment and that push-offs (time B) were not registered as response. Data from x- and y-coordinates of the COP were sampled at a rate of 33 Hz.

### Statistical analysis

Growth curve modeling [12] or hierarchical linear modeling [13], are both generalizations of standard regression approaches. The major difference between a growth curve model and standard regression model is that a growth curve model contains two hierarchically related sub models, rather than a single model that applies to the entire sample. The first sub model, usually called level-1, captures the effect of time. The level-1 model gives a value for a dependent variable Y, for a participant *i*, at a measurement occasion *j*. Let 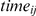 be the linear effect of time, and let 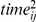, 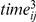 and 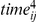 be the power polynomials indicating the quadratic, cubic and quadric effects.

The regression function for the first level is defined as follows:
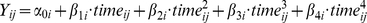
As in standard regression models this level 1 equation has an intercept term 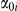, and predictor effect 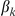 for each predictor *k* and an error term 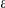. However, unlike standard models, the intercept and the predictor effects contain a subscript *i* and therefore are allowed to vary across individuals *i*. This variation is captured in the second set of models, called level-2 models. That is, there may be a level-2 model for each parameter of the level-1 model, which describes that level-1 parameter in terms of population means, fixed effects, and random effects. In our study, the starting position of all participants was fixed at time 0 and their subsequent movements were measured relative to this fixed position. Therefore, the intercept 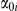 was fixed to be 0. For each of the effects of the four polynomials we defined a level 2 submodel:
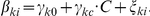
In this model parameter 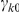 estimates the value of the polynomial term when all other terms (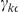 and 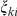) in its particular level-2 model are zero. Parameter 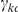 estimates the effect of condition (forward, backward) on the polynomial term. Parameter 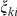, finally, is the error term that allows for individual variation around these effects. In total the model contains four parameters 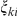 for each of the polynomial terms. We allowed these random effects to be correlated so six parameters were estimated for the correlations between the four random effects.

The program Latent Gold 4.0 [14] was used to estimate parameters and calculate the fit of the models. A standard significance test for adding a parameter to a model is the deviance statistic -2LL (minus 2 times log-likelihood). Change in deviance in log-likelihood, ΛD, is distributed as chi-square, with degrees of freedom equal to the number of parameters added. The change in deviance allows us to test whether including parameter increases the fit of the model.

## Results

### Accuracy

Accuracy ratings for the sensibility judgment task were recorded for each participant. No subjects were removed from the analysis based on accuracy. In the standing version of the experiment mean accuracy was 98% with a minimum of 93% and a maximum of 100% (SD = 2.3). Mean accuracy for the sitting version was 99% with a minimum of 92% and a maximum of 100% (SD = 2.5). Incorrect trials were removed from further analysis.

### Response Times

To check for the comparability of forward and backward sentences, a paired-samples *t*-test was done between response times on forward and backward sentences on the overall data and for each experiment separately. Response times were defined as the time between the start of a trial and time C in Figure 2. No difference in response times was found between forward and backward sentences in the standing version [*t* (7) = 0.26, *p*>.79] nor in the sitting version [*t* (7) = 1.79, *p*>.11]. Therefore, we assumed that both types of critical sentences were equally difficult.

### Growth curve analysis balance data

We aligned the y-axis curves in time at response offset (time D in Figure 2) in order to compare and analyze the response curves and so that the starting point of each curve was equal to zero on the y-axis, because differences in intercept could be the result of different starting postures. This way balance shifts on the y-axis can only be interpreted in terms of differences between conditions and shifting balance more forward or backward, but not in terms of a concrete position of the COP.

We performed separate growth curve analyses for the standing version and the sitting versions of the experiment. For each version we calculated different kinds of models. Model 1 is a baseline model that includes the four polynomial effects but does not include the effect of condition. Model 2 has the same terms as the baseline model but in addition has an effect of condition on the linear term is. Model 3 is equal to model 2 but in addition has an effect of condition on the quadratic term. Model 4 is equal to model 3 but in addition has an effect of condition on the cubic term. Model 5, finally is the baseline model and in addition contains an effect of condition on all polynomial terms.

First, we compared the fit of the baseline model 1 with model 2, which in addition contains an effect of condition in the linear term. The *p*-value of the chi-squared significance test on the ΛD with 1 degree of freedom is significant (p<.001) indicating that adding a parameter for condition on the linear term significantly improved the fit. The difference in fit between model 2 and model 3, in which the parameter for condition on the quadratic term was added was also significant (p = .009). The results of the analysis are summarized in Table 2. The difference between the fit of model 3 and model 4 was not significant nor was the improvement in fit from model 4 to model 5. We therefore conclude that model 3, which contains a linear and quadratic effect for condition fitted the data best.

**Table 2:** Summary of the growth curve analyses for the standing and sitting experiments.

Standing				
Model	Par	LL	ΛD	*p*
1. Time polynomials	15	1165.27	-	-
2. Previous model+condition linear	16	1203.71	38.44	<.001
3. Previous model+condition quadratic	17	1207.18	3.47	.009
4. Previous model+condition cubic	18	1207.83	0.65	.25
5. Previous model+quadric condition	19	1208.20	0.47	.33

Sitting				
Model	Par	LL	ΛD	*p*
1. Time polynomials	15	1341.11	-	-
2. Previous model+condition linear	16	1367.32	26.21	<.001
3. Previous model+condition quadratic	17	1374.84	7.52	<.001
4. Previous model+condition cubic	18	1379.56	4.72	.002
5. Previous model+quadratic condition	19	1379.60	0.04	.777

**Figure 3:**
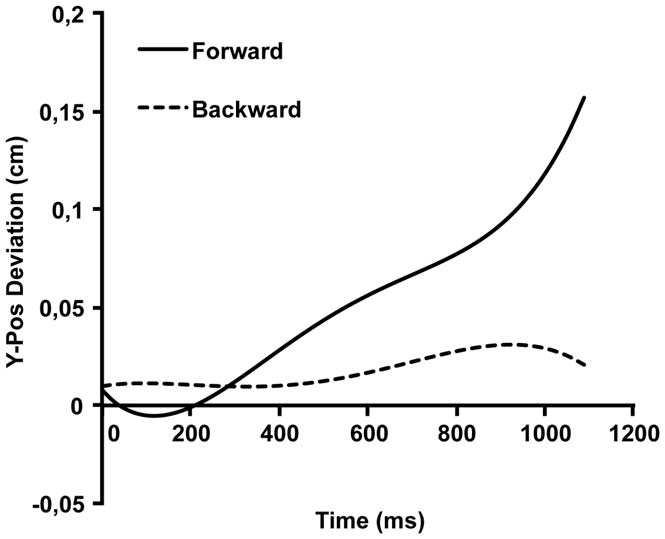
Estimated averaged response curves of y-axis movements from x-axis response onset to response offset in the standing condition.

Figure 3 shows the estimated averaged curves of the y-coordinates of the COP for forward and backward sentences in the standing condition according to model 2. The key finding is a linear effect of condition. Overall, the forward condition has produced a more forward trajectory than the backward condition. This effect increases over the course of the response, which is due to the quadratic effect.

**Figure 4:**
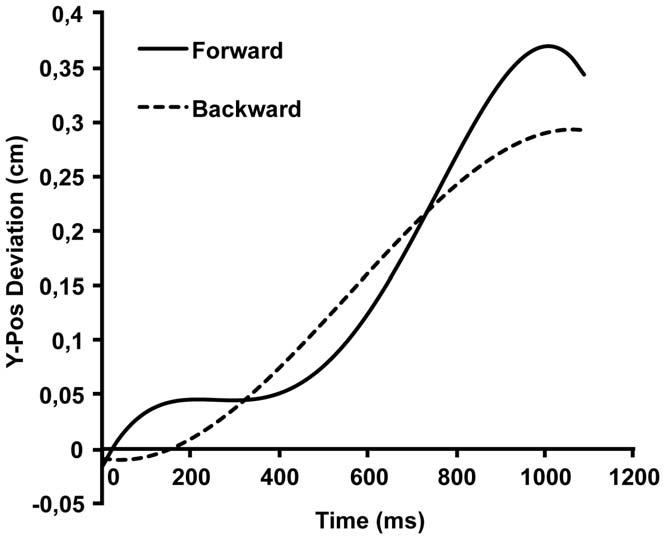
Estimated averaged response curves of y-axis movements from x-axis response onset to response offset in the sitting condition.

Figure 4 shows the averaged curves for the y-coordinate of the COP in the forward and backward condition for the sitting version (notice the difference in scale on the y-axis with Figure 3). The results are summarized in Table 2. The key finding is once more the linear effect of condition; the forward sentences produced more forward movement than the backward sentences. In addition, there is a cubic effect, which shows that this effect first becomes larger and then smaller over the course of the response. We assume that the cubic effect is due in part to the biomechanics of moving sideways while seated.

## Discussion

Responding to sentences describing actions implying a forward or backward balance shift modulates anterior-posterior balance shifts in a congruent way in participants regardless of the response direction (sideways instead of forward or backward) and body position of the reader (sitting or standing). The linear effect visible in Figures 3 and 4 is consistent with this interpretation. Results of the standing condition show roughly the same effects as the sitting condition. In addition to the linear effect, there was a quadratic effect, which was not anticipated from the theoretical viewpoint of motor resonance. We have no explanation for this effect but assume it arises because the ACE interacts with the biomechanics of moving sideways, which differs between the standing and the sitting conditions. It is interesting to note that the effect along the y-axis is larger when subjects are seated than when they are standing. In a seated position, the legs can be used as a counterweight, allowing more movement of the upper body.

The current study builds on the large number of studies investigating motor resonance during language comprehension, specifically on those that have investigated the ACE. As we mentioned in the introduction, action compatibility is not an all-or-none phenomenon. Rather, actions may be compatible on any number of dimensions. In the original ACE experiments, the actions described in the sentences were largely consistent with the actions performed by the subjects to respond to the sentences. But even in those experiments, the overlap was not perfect. For example, we typically have our hand upside down (palm up) when we open a drawer, but in Glenberg and Kaschak's experiments [8], subjects typically had their palm down when pressing the buttons. Similarly, we typically have our hand upside down when screwing in a light bulb, but Zwaan and Taylor's subjects had their hand right side up [9]. The main source of overlap was direction relative to the body in the Glenberg and Kaschak experiments and rotation direction in the experiments by Zwaan and his colleagues. In our experiments here, the response direction—presumably the most salient part of the response—was orthogonal to the described movement. Moreover, the performed movement was incompatible with the described movement; moving sideways does not land you into the pool. Despite this fact, the direction of the described action modulated the response action. This was even the case when the subject's posture was also incompatible with the described action. You cannot sit down when you are already seated. It could be argued that we already know this from neuroimaging studies that have found motor activation in sentence comprehension [15]–[17]. Lying in a scanner is of course also posturally inconsistent with kicking a ball. However, in those experiments the subjects were instructed to lie still, whereas in the current experiments, they were instructed to make an incongruous movement as well.

So what do we make of the fact that the ACE persisted despite these action incompatibilities? Our interpretation of the fact that sentence content modulated response is that understanding the sentence involved activating a forward or backward movement direction, which was integrated with the response movement our subjects made—from left to right—to produce the curves we examined. Evidently, it is not necessary for the described action to have a great deal of overlap with the performed action to observe these effects. The overt response apparently does not have a “lock” on the motor system. It can be penetrated by the results of language comprehension, even if this involves descriptions of seemingly incompatible actions. The ACE, in other words, is a very robust phenomenon.

We should not take these results to mean that motor resonance is necessarily involved in comprehending all kinds of actions. The actions that we have investigated here are all relatively simple and punctate ones. It is difficult to imagine what kind of motor resonance would occur with sentences like *He is building a house* or *He is playing baseball*. Moreover, neuroimaging studies suggest that figurative sentences, such as *He kicked the bucket* do not produce activation in motor areas of the brain, whereas their literal counterparts do [16]–[17]. These results suggest that only when the described action is a concrete one does motor resonance occur. Other behavioral results suggest that there are limitations to concrete actions as well. If actions are described as plans to act (*He was about to start the car*), motor resonance does not occur, whereas it does occur if the action is being performed concurrently or has been performed in the past [18]. An integrative account of this accumulation of results is that motor resonance occurs if the situation model contains a concrete punctate action, but is perhaps limited to these cases. The present results indicate that motor resonance may occur in such cases even if there are substantial incompatibilities between the described and performed actions. In this sense we agree with the observation that “sensory-motor representations may contribute to … the building of detailed situation models” [5], [19].
